# The Use of Whole Exome Sequencing in a Cohort of Transgender Individuals to Identify Rare Genetic Variants

**DOI:** 10.1038/s41598-019-53500-y

**Published:** 2019-12-27

**Authors:** J. Graham Theisen, Viji Sundaram, Mary S. Filchak, Lynn P. Chorich, Megan E. Sullivan, James Knight, Hyung-Goo Kim, Lawrence C. Layman

**Affiliations:** 1 Section of Reproductive Endocrinology, Infertility, & Genetics, Department of Obstetrics & Gynecology, Medical College of Georgia, Augusta University, Augusta, Georgia United States; 2 Section of Reproductive Endocrinology & Infertility, Department of Obstetrics & Gynecology, University of California, San Francisco, San Francisco, California United States; 3 Department of Genetics, Yale University School of Medicine, New Haven, Connecticut United States; 4 Yale Center for Genome Analysis, Yale University, New Haven, Connecticut United States; 5 Present Address: Neurological Disorders Research Center, Qatar Biomedical Research Institute, Hamad Bin Khalifa University, Doha, Qatar

**Keywords:** Development, Genomics

## Abstract

Approximately 0.5–1.4% of natal males and 0.2–0.3% of natal females meet DSM-5 criteria for gender dysphoria, with many of these individuals self-describing as transgender men or women. Despite recent improvements both in social acceptance of transgender individuals as well as access to gender affirming therapy, progress in both areas has been hampered by poor understanding of the etiology of gender dysphoria. Prior studies have suggested a genetic contribution to gender dysphoria, but previously proposed candidate genes have not yet been verified in follow-up investigation. In this study, we expand on the topic of gender identity genomics by identifying rare variants in genes associated with sexually dimorphic brain development and exploring how they could contribute to gender dysphoria. To accomplish this, we performed whole exome sequencing on the genomic DNA of 13 transgender males and 17 transgender females. Whole exome sequencing revealed 120,582 genetic variants. After filtering, 441 variants in 421 genes remained for further consideration, including 21 nonsense, 28 frameshift, 13 splice-region, and 225 missense variants. Of these, 21 variants in 19 genes were found to have associations with previously described estrogen receptor activated pathways of sexually dimorphic brain development. These variants were confirmed by Sanger Sequencing. Our findings suggest a new avenue for investigation of genes involved in estrogen signaling pathways related to sexually dimorphic brain development and their relationship to gender dysphoria.

## Introduction

Gender dysphoria occurs when an incongruence between an individual’s expressed and assigned gender causes them to experience clinically significant distress or functional impairment^1^. Over the last few decades, estimates of the prevalence of gender dysphoria have been rising, most recently suggesting that, in adults, gender dysphoria is experienced by 0.5–1.4% of natal males and 0.2–0.3% of natal females^2^. The related term, transgender identity, is importantly not synonymous with gender dysphoria. Though both terms include individuals whose natal sex and expressed gender are not congruous, a person experiencing gender dysphoria may not necessarily self-describe as transgender, and a transgender individual may no longer experience gender dysphoria after taking steps to express themselves in ways more consistent with their expressed gender^3^. The methods, timing, and extent of these steps, a process commonly known as “transition”, are unique to each person and range from socio-behavioral changes to hormonal and surgical therapies. Thus, a person initially experiencing gender dysphoria may experience improvement or even complete resolution of that dysphoria as they progress through the self-actualizing process of transitioning to become a transgender person.

Despite the well-documented mental and physical health benefits associated with transitioning, transgender individuals do face several unique challenges in their daily lives. They experience increased rates of societal discrimination and sexual violence and are at increased risk for depression, substance abuse, and attempted suicide^4,5^. Likely due to stressors such as these, twenty-six percent of surveyed transgender individuals reported the use of alcohol or drugs as a coping mechanism^6^.

In addition to societal obstacles, transgender individuals also face unique challenges with regard to obtaining healthcare. Nineteen percent of transgender individuals reported being denied service by a physician or other medical provider because of their gender identity, and more than a quarter of these patients report prior verbal harassment in a medical environment^6^. Further, insurance companies do not reliably cover the costs of gender affirming hormone or surgical therapies, even though these therapies are identified as both cost effective and medically necessary for many transgender individuals^7–21^.

One factor contributing to the above obstacles is an overall lack of understanding regarding the biologic basis of gender dysphoria, though studies have suggested a genetic contribution. In particular, multiple studies have compared concordance rates of gender dysphoria in monozygotic and dizygotic twin pairs in order to estimate its heritability (i.e. the proportion of gender dysphoria that can be ascribed to genetic variation). In adolescents, these studies reported heritability estimates between 38–47% in natal females and 25–43% in natal males, while in adults, estimates ranged between 11–44% and 28–47% respectively^22^. One exception, a study conducted in Japan by Sasaki *et al*., evaluated 4354 twin pairs and showed no heritable component to gender dysphoria in adolescent or adult natal males^23^. However, this study did not achieve statistical significance, making it difficult to infer conclusive meaning from the findings. Though most molecular genetic studies in this area have been limited by small sample size and lack of reproducibility, several genetic variants have been proposed as candidates based both on their rarity in the population and their biologic plausibility^24,25^. These include variants of *COMT, RYR3*, SRD5A2, STS, and SULT2A1 as well as variants of genes coding aromatase, androgen receptor, estrogen receptors α & β, and 17α-hydroxylase^24–27^. In our study, we sought to contribute to these findings and introduce a new framework for candidate variant selection, based on current models of sexually dimorphic brain development.

In contrast to the limited knowledge regarding the development of human gender identity, there has been significant progress, over the last twenty years, using animal models to demonstrate the neurodevelopmental pathways leading to sexually dimorphic brain regions and resultant sex-specific behavior patterns^28–33^. In rodents, four key areas of the brain have been identified with developmental pathways leading to sexual dimorphism: the ventromedial nucleus (VMN), the medial preoptic area (mPOA), the anteroventral periventricular nucleus (AVPV), and the arcuate nucleus. In each of these regions, the identified dimorphic pathways are initiated by neuronal estrogen receptor (ER) activation and result in sex-specific regional differences in dendritic density or volume.

In this study, we identify rare variants in genes associated with these pathways of sexual differentiation in the brain and explore how they could contribute to gender dysphoria and transgender identity. To accomplish this, we performed whole exome sequencing (WES) on the genomic DNA of 30 unrelated transgender individuals.

## Methods

### Patients

This study was approved by the Augusta University Institutional Review Board, and the methods were carried out in accordance with the principles stated in the American Society of Human Genetics Code of Ethics. Patients presenting to the Equality Clinic of Augusta or from the Augusta University Reproductive Endocrinology, Infertility, and Genetics Clinic for gender affirming hormone therapy were enrolled. Subjects were eligible for enrollment if they were at least 18 years of age, had been diagnosed by a physician with gender dysphoria based on DSM-V criteria (Supplemental Table 1) and self-identified as a transgender individual. Written informed consent was obtained from all participants prior to enrollment.

### Whole exome sequencing

Genomic DNA was extracted from the blood of all enrolled subjects and treated with RNAse to remove residual RNA. DNA (2–3 μg/subject) was sent to the Yale Center for Genome Analysis for WES. DNA was sheared to a mean fragment length of 220 bp using focused acoustic energy (Covaris E220; Woburn, Massachusetts). Fragments were then blunt ended and phosphorylated using T4 DNA polymerase and T4 polynucleotide kinase. Custom adapters were ligated to each fragment using T4 DNA ligase before DNA fragments were PCR amplified.

The amplified DNA was then heat-denatured and mixed with biotinylated DNA probes (IDT xGen Exome Panel; Coralville, Iowa). Hybridizations were performed at 65 °C for 16 hours. The captured fragments were PCR amplified and then purified with AMPure XP beads to generate each subjects’ DNA library. The Illumina NovaSeq. 6000 S4 platform was used to perform paired-end sequencing on these DNA libraries, with reads of 2 × 100 bp. Burrows-Wheeler Aligner (BWA) was used to map sequence reads to the genome. The Yale Exome Pipeline was used to call exome-wide variants, and Annovar and Variant Effect Predictor were used for variant annotation.

### Filtering of variants

**Figure 1:**
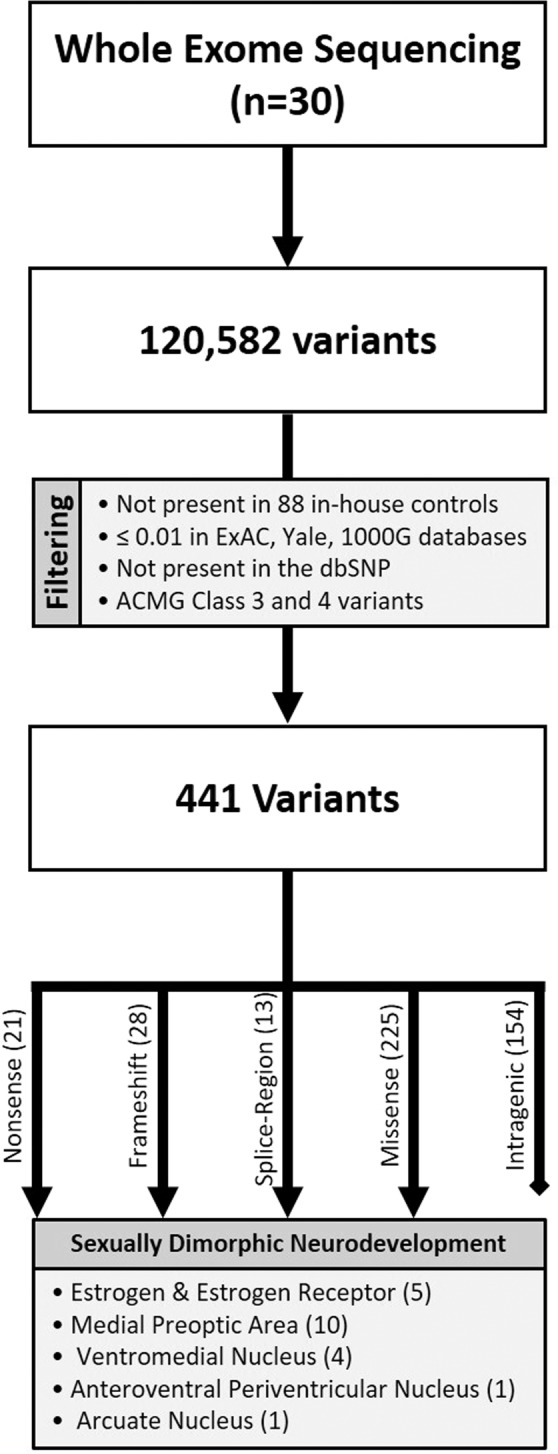
Flowchart for Selection of Candidate Genes.

Annotated variants were filtered using the following criteria: 1) not present in 88 in-house control exomes from non-transgender individuals (17 males and 71 females), 2) frequency less than 0.01 in the ExAC, 1000 Genome, and Yale databases, 3) not listed in the dbSNP, 4) ACMG Class 3 and 4 variants: includes frameshift, splice-site (intronic variants occurring 1-2 bps from the intron/exon junction), splice-region (exonic or intronic variants occurring 1-3 bps or 3-8 bps from the intron/exon junction respectively), nonsense variants as well as missense variants with Combined Annotation Dependent Depletion (CADD) score ≥20 (Fig. 1). Variants with a CADD ≥20 are predicted to be in the top 1% of those most likely to affect the function of a given gene when compared to wild-type^34^.

### Selection of candidate genes for sanger sequencing

After filtering, candidate variants were selected for Sanger Sequencing confirmation if they involved genes related to pathways of sexually dimorphic brain development. Because sex-specific brain development is still poorly understood in humans, we began by performing a thorough literature review of prior research involving sexually dimorphic brain development in animal models (Supplementary Fig. 1). We then focused our analysis on variants of genes associated with those developmental pathways that had the potential to be conserved in humans. The selection process was as follows: (1) Data was collected regarding the descriptions, known functions, and ontologies of all genes involved by the variants that remained after the initial filtering process. Three databases were used to collect this data including NCBI Reference Sequence, Gene Ontology Consortium, and Online Mendelian Inheritance in Man (OMIM)^35–38^; (2) the information gathered from these databases was compared to known pathways of sexually dimorphic brain development in rodent models^28,33^; (3) genes were selected as candidates if their descriptions, functions, or ontologies suggested an association with these pathways; (4) variants that both remained after filtering *and* involved the above candidate genes were selected for confirmation via Sanger Sequencing.

### Sanger sequencing

In each instance, DNA primers were selected using NCBI Primer-BLAST. Amplification of DNA was performed using polymerase chain reaction under the following conditions: samples were first heated to 95 °C for 5 minutes to allow DNA denaturation. They were then subjected to 30 cycles of the following conditions to allow primer annealing: one minute at 95 °C, 30–60 seconds at 55 °C, and 30–60 seconds at 72 °C. Finally, samples were heated to 72 °C for 7 minutes to allow DNA extension. After PCR amplification, the presence of DNA fragments of the anticipated size was confirmed with agarose gel electrophoresis, visualized under UV light. Confirmed PCR products were then subjected to ethanol precipitation, before and after a sequencing reaction was performed. Products of the sequencing reaction were then analyzed with an ABI 310 Sequencer and individually confirmed by the investigators.

## Results

For this preliminary study, 30 transgender individuals were enrolled, including 13 transgender males (i.e. natal females transitioning to male) and 17 transgender females (i.e. natal males transitioning to female). WES with a coverage of 98.36% and average read depth of 75 demonstrated 120,582 genetic variants. After filtering, 441 variants in 421 genes remained for further consideration. This included 21 nonsense, 28 frameshift, 13 splice-region, and 225 missense variants. The remaining 154 intragenic variants appeared in non-coding regions of the genes, defined as the 5′UTR, 3′UTR or intronic variants located >8 bp away from a splice junction. These variants from non-coding regions were not considered for further analysis. From the 421 genes that remained after filtering, 19 were found to have associations with pathways of sexually dimorphic brain development and were thus selected as candidate genes. Twenty-one variants were noted in these candidate genes and confirmed with Sanger Sequencing.

### Nonsense variants

After filtering, twenty-one nonsense variants, of those originally identified by WES, remained (Table 1). With the exception of a variant of *DIAPH2*, which is localized to the X chromosome, each variant was observed in only a single subject, and all were heterozygous. The variant *DIAPH2*, c.G736T (p.G246X), was noted in three transgender males and one transgender female, with the transgender female reported as hemizygous and the transgender males reported as heterozygous.

**Table 1:** Nonsense Variants Called by Whole Exome Sequencing.

Gene	Location	Position^^^	Nomenclature	Het^◊^ (n)	Hom^□^;(n)
ACRBP	12p13.31	6753324	c.G923A (p.W308X)	1	0
AGAP5	10q22.2	75435544	c.C874T (p.R292X)	1	0
AIM1	6q21	106968165	c.C1858T (p.Q620X)	1	0
C1R	12p13.31	7187951	c.C2062T (p.W244X)	1	0
CEP131	17q25.3	79173533	c.C1009T (p.R337X)	1	0
COL17A1	10q25.1	105806508	c.C2359T (p.Q787X)	1	0
CTC1	17p13.1	8137838	c.C1753T (p.Q585X)	1	0
CTNNA2	2p12	80136894	c.C1027T (p.Q343X)	1	0
DIAPH2	Xq21.33	96171440	c.G736T:p.(G246X)	3	1^*^
FAM186A	12q13.12	50744311	c.G6304T (p.E2102X)	1	0
GSS	20q11.22	33519849	c.C922T (p.Q308X)	1	0
INHBE	12q13.3	57850008	c.C430T (p.R144X)	1	0
KIAA1109	4q27	123245675	c.C10888T (p.R3630X)	1	0
MUC16	19p13.2	9012485	c.C38721A (p.C12907X)	1	0
NUP210L	1q21.3	154067536	c.C2062T (p.R688X)	1	0
OR1E2	17p13.2	3336880	c.C256T (p.Q86X)	1	0
PIK3CA	3q26.32	178917534	c.C409T (p.Q137X)	1	0
PPARGC1B	5q32	149109981	c.C76T (p.Q26X)	1	0
TAS1R3	1p36.33	1268013	c.C1102T (p.Q368X)	1	0
TEP1	14q11.2	20854317	c.C2899T(p.Q967X)	1	0
TNN	1q25.1	175054558	c.G1252T(p.E418X)	1	0

^^^Position based on GRCh37.p13 Primary Assembly ^*^Reported as hemizygous in a transgender female subject ^◊^Heterozygous ^□^Homozygous.

### Frameshift variants

Of the frameshift variants identified by WES, there were 28 that remained after filtering (Table 2). In general, all frameshift variants were heterozygous, including a variant of *PPP2R3B*, c.356dupC (p.P119fs), located on chromosome X, in one transgender male. One exception to this was noted in a single transgender male, who was homozygous for a variant of *PRAMEF13*, c.1291_1292insA (p.A431fs).

**Table 2:** Frameshift Variants Called by Whole Exome Sequencing.

Gene	Location	Position^^^	Wild-Type	Variant	Protein Δ	Het^**◊**^ (n)	Hom^**□**^; (n)
ABCB4	7q21.12	87069011	TCACTTTCTGTGTC	T	p.D564fs	1	0
ADAMTS15	11q24.3	130319306	G	GGCGCA	p.A146fs	1	0
BIRC6	2p22.3	32726827	TTGGATGCCATATCAGTTGGGGA	T	p.L3027fs	1	0
CAPN2	1q41	223938640	TGG	T	p.W288fs	1	0
EPG5	18q12.3-q21.1	43493697	C	CAGAGTTTATCACCAATTCCCCTTCAATA	p.A1264fs	1	0
ERICH2	2q31.1	171638777	CA	C	p.A249fs	1	0
FASTKD1	2q31.1	170428515	CT	C	p.L8fs	1	0
GAL3ST4	7q22.1	99758165	A	AC	p.S283fs	1	0
HIST1H2AK	6p22.1	27805753	TCA	T	p.T121fs	1	0
IGSF10	3q25.1	151166309	AC	A	p.V487fs	1	0
KCNJ12	17p11.2	21319185	TG	T	p.G178fs	1	0
KIAA1919	6p22.3	111587872	TC	T	p.P370fs	1	0
KRT19	17q21.2	39680651	C	CAGCA	p.E268fs	1	0
METTL17	14q11.2	21458654	G	GC	p.V87fs	1	0
MYOF	10q23.33	95132692	GA	G	p.F817fs	1	0
NPIPB15	16q23.1	74411878	C	CT	p.L3fs	1	0
NUMB	14q24.2-q24.3	73743337	A	AGGGGAGGGATTAGTAC	p.T588fs	1	0
OR2T1	1q44	248604696	G	GT	p.Y64fs	1	0
PPP2R3B	Xp22.33	322293	C	CG	p.P119fs	1	0
PRAMEF13	1p36.21	13448183	G	GT	p.A431fs	0	1
RNF213	17q25.3	78269608	T	TC	p.P669fs	1	0
SLC44A4	6p21.33	31833803	TAGATTTGC	T	p.L442fs	1	0
SPO11	20q13.31	55917787	TA	T	p.L321fs	1	0
TADA1	1q24.1	166838708	C	CGCGTGAGAATGGCCAGGAGGAAATCATTGTGAGA	p.R69fs	1	0
TBP	6q27	170871045	A	AGC	p.Q74fs	1	0
TTF2	1p13.1	117632763	GTA	G	p.S810fs	1	0
UHRF1BP1	6p21.31	34839648	GC	G	p.P1382fs	1	0
ZNF806	2q21.2	7585345	C	CAG	p.K51fs	1	0

^^^Position based on GRCh37.p13 Primary Assembly ^◊^Heterozygous ^□^Homozygous.

###  Splice-region variants

Of the splice-region variants initially identified by WES, 13 remained after filtering (Table 3). All splice-region variants were heterozygous and noted in only a single subject.

**Table 3:** Splice Site Variants Called by Whole Exome Sequencing.

Gene	Location	Position^^^	Wild-Type	Variant	Protein Δ	Het^◊^ (n)	Hom^□^ (n)
BACH2	6q15	90660300	G	A	p.R509C	1	0
CYP2D6	22q13.2	42522855	G	T	p.A438E	1	0
DUS1L	17q25.3	80022002	C	T	p.G116S	1	0
EPB41L1	20q11.23	34807685	C	G	p.I606M	1	0
FBXO11	2p16.3	48035327	G	C	p.P821R	1	0
KDM6B	17p13.1	7756696	C	G	p.L1636V	1	0
MRTO4	1p36.13	19584973	G	A	p.V166M	1	0
OR2H1	6p22.1	29429779	A	G	p.Q78R	1	0
PIBF1	13q22.1	73409504	C	A	p.N407K	1	0
PRRC2B	9q34.13	134308006	A	T	p.I40F	1	0
RELN	7q22.1	103202398	G	A	p.S1738F	1	0
TBK1	12q14.2	64891430	A	T	p.L654F	1	0
TMPRSS11E	4q13.2	69344567	G	T	p.G323V	1	0

^^^Position based on GRCh37.p13 Primary Assembly ^◊^Heterozygous ^□^Homozygous.

### Missense variants

The majority of variants present after filtering were missense variants with a CADD score ≥ 20. In total, 225 variants were noted involving 213 genes (Supplemental Table 2). Variants in this group were heterozygous and only observed in a single individual, unless otherwise noted below. A variant of PATL1, c.C728T (p.S243F), was shared by two transgender females. A variant of *RASA4B*, c.G1988A (p.R663Q), was shared by two transgender males and one transgender female. Two variants in *DSCAM*, c.C5411A (p.T1804K) and c.G5413A (p.E1805K), were found concomitantly in two transgender females and one transgender male. A variant of APBB1IP, c.C1040A (p.T347N) was noted in two transgender males and one transgender female. One transgender male and one transgender female shared a variant of *GOLGA8J*, c.C931G (p.L311V), another transgender male and female pair shared a variant of *FCGBP*, c.G9790A (p.G3264S), and a variant of GGT5, c.T602C (p.L201P), was noted in a final transgender male and transgender female pair. Finally, one transgender male was homozygous for a variant of *FAM230B*, c.A1484C (p.Val495Gly).

When specifically considering missense variants of genes located on the sex chromosomes, in transgender females there were seven hemizygous variants noted in six genes located on the X chromosome: *ASMT, CXORF57, GTPBP6, P2RY8, PLCXD1*, and *RGAG1*. None of these genes has been shown to escape X-linked inactivation^39^. All but one variant was noted in only a single individual. Two transgender females were noted to have two concomitant hemizygous *PLCXD1* variants, c.T512C (p.I171T) and c.G295A (p.G99R). In transgender males, four variants were noted in genes on the X chromosome: *ATRX, GTPBP6, PPP2R3B*, and *ZXDA*. All of these were heterozygous and observed in only a single subject. After filtering, there were no remaining variants of genes located on the Y chromosome.

### Genes related to pathways of sexual differentiation in the brain

The variants described above were compared to the previously described pathways of sexual differentiation in the brain, and 21 variants involved in 19 genes related to these pathways: *AKR1C3, BOK, CDH8, CDK12, CTNNA2, DNER, DSCAML1, EGF, EFHD2, GRIN1, KCNK3, MAP4K3, PIK3CA, PPARGC1B, RIMS3, RIMS4, SPHK1, SYNPO*, and *TNN* (Table 4). These included 17 missense and 4 nonsense variants, and each was confirmed via Sanger Sequencing. Each of these variants was heterozygous found in a single individual.

**Table 4:** Variants of Candidate Genes Related to Sexual Differentiation in the Brain.

Relation	Gene	Location	Position^^^	Nomenclature	Description
Estrogen & Estrogen Receptor	AKR1C3	10p15.1	5144369	c.A647G (p.Y216C)	AKR1C3 is capable of metabolizing estrogen and progesterone and has been shown to possess 11-ketoprostaglandin reductase activity in metabolizing prostaglandins
	CDK12	17q12	37618361	c.G37C (p.G13R)	Silencing CDK12 in human breast cancer cell lines caused resistance to inhibition of estrogen signaling
	PIK3CA	3q26.32	178917534	c.C409T (p.Q137X)	PIK3CA inhibition mediates an open chromatin state at the ER target loci in breast cancer models and clinical samples
	PPARGC1B	5q32	149109981 149213068	c.C76T (p.Q26X) c.C1315T (p.R439C)	PPARGC1B is a selective coactivator of estrogen receptor alpha
Medial Preoptic Area	SPHK1	17q25.1	74383224	c.C712T (p.L238F)	SPHK1 modulates COX2 acetylation in neurons using acetyl-CoA and likely has acetyl-CoA-dependent cytoplasmic acetyltransferase activity towards COX2
	DNER	2q36.3	230578982	c.G158T (p.C53F)	1. DNER mediates neuron-glia interaction and promotes morphological differentiation of Bergmann glia through Deltex-dependent Notch signaling 2. Ca2+-permeable AMPA receptors on glia are required for proper structural and functional relationships between Bergmann glia and glutamate-mediated synapses in the cerebellum.
	CDH8	16q21	61858936	c.A815G (p.N272S)	CDH8 is enriched at glutamatergic synapses on cortical neurons and in striatum, and its knockdown impairs dendritic arborization
	CTNNA2	2p12	80136894	c.C1027T (p.Q343X)	In homozygous CTNNA2 mutants, neuronal overexpression of alpha-N-catenin resulted in restoration of normal spine morphology, whereas in wild-type cells, overexpression of alpha-N-catenin resulted in significantly increased synaptic density and dendritic spine density
	DSCAML1	11q23.3	117340673	c.G3157A (p.V1053I)	IgSF members (such as DSCAMs) participate in determining the inner plexiform layer sublaminae in which synaptic partners arborize and connect. Thus vertebrate DSCAMs play roles in neural connectivity
	EGF	4q25	110890223	c.G1546C (p.E516Q)	EGF has been shown to stimulate tyrosine phosphorylation of focal adhesion kinase and paxillin, which have been implicated as regulators of sex differences in neuronal morphology
	EFHD2	1p36.21	15753774	c.G585T (p.E195D)	Knockdown of EFHD2 in cultured mouse cortical neurons resulted in synapse number loss, but not alteration of neurite outgrowth, suggesting that EFHD2 is involved in control of synapse development and maintenance
	SYNPO	5q33.1	150028774	c.G937A (p.G313R)	Homozygous deletion of SYNPO in mice resulted in complete lack of dendritic spine apparatuses accompanied by a reduction in long-term potentiation in affected areas of the hippocampus
	TNN	1q25.1	175054558 175046693	c.G1252T (p.E418X) c.A139G (p.K47E)	TNN mediates specific repulsive properties on neurites and neurons
The Ventromedial Nucleus	RIMS3	1p34.2	41107405	c.A193G (p.S65G)	RIMS3 and RIMS4 belong to the RIM protein family that function as important components of the presynaptic machinery for synaptic vesicle fusion and neurotransmitter release
	RIMS4	20q13.12	43384843	c.C745T (p.P249S)	
	GRIN1	9q34.3	140036549	c.C343G (p.R115G)	NMDA receptor (GRIN1) activation may participate in neuronal growth and/or anti-apoptosis, and support an important signaling pathway of NF-κB activation and its target gene, Bcl-2, in preventing neuronal apoptosis in the SDN-POA of male rats during sexual development
	MAP4K3	2p22.1	39499479	c.C1855G (p.Q619E)	Calcium influx through the NMDA receptors leads to activation of MAP kinase, resulting in transcription of genes associated with the construction and maintenance of dendritic spines.
AVPV^◊^	BOK	2q37.3	242501871	c.C329T (p.A110V)	BOK induces cytochrome-c release and apoptosis in the absence of both BAK and BAX. Modulation of endogenous Bok levels affects the apoptosis response
Arcuate Nucleus	KCNK3	2p23.3	26950997	c.T746C (p.M249T)	Neuronal KCNK3 is upregulated in a compensatory fashion in response to GABAAR disruption

All individual variants confirmed by Sanger sequencing were heterozygous and found in a single subject ^^^Position based on GRCh37.p13 Primary Assembly ^◊^Anteroventral Periventricular Nucleus.

## Discussion

The factors that lead to gender dysphoria are poorly understood, but given that prior research has suggested a significant heritable component, we sought to explore what the possible genetic contribution could be^22^. Somewhat counterintuitively, the ER initiated developmental pathways occurring in the VMN, mPOA, AVPV, and the arcuate nucleus are active in natal males rather than in natal females^33^. This is because, during the perinatal period, the testes initiate a rapid but transient surge in serum testosterone, which can readily be aromatized to estradiol, thus driving the ER activated neurodevelopmental pathways that result in male pattern behavior^33,40,41^. Conversely, during this same perinatal period, the ovaries are quiescent in natal females, and the resulting lack of ER stimulation drives sex-specific neurodevelopment toward the path of feminization^33^.

In a series of animal model studies confirming these pathways, researchers exposed developing rodents to substrates that would either induce ER activated pathways in females (i.e. exposure to testosterone or estradiol) or disrupt those pathways in males. In each of these models, investigators were able to induce cross-sex neurodevelopment and resulting cross-sex behavior in rodents, scored by examination of lordosis and proceptive behavior in males and by mounting and thrusting events in females^42^. Interestingly, despite the fact that cross-sex behavioral changes only manifested at sexual maturity, they only occurred if neurodevelopmental alterations were induced during the perinatal period^28^. This suggested that sex-specific neurodevelopment must be initiated during a short perinatal window, but that the resulting behavioral effects only emerge with the awakening of the hypothalamic-pituitary-gonad axis (i.e. puberty). This short span of neurodevelopmental malleability was dubbed the “critical period”, and it corresponds to the spontaneous surge in testosterone production that occurs in natal males^33^. Of note, similar patterns have been observed in at least nine other mammalian species including non-human primates^41^. Overall, the above evidence suggests that, in many species and by proxy of estrogen, testosterone’s presence or absence during the perinatal period drives diverging pathways of permanent sex-specific neurodevelopment, which later result in sex-specific behavior.

Though sex-specific brain development has not yet been thoroughly evaluated in humans, the above pattern is consistent with the average developmental timeline of gender dysphoria, which often significantly worsens at the time of puberty, and there is ample evidence to suggest that sex-hormone exposure in the prenatal period does affect sex-specific behavior in humans. The preponderance of data has been obtained through research involving natal females with classic congenital adrenal hyperplasia. These individuals are exposed to elevated levels of androgens prior to birth, with varying degrees external genital virilization. Several studies evaluating behavior patterns in natal female children with congenital adrenal hyperplasia have demonstrate that they are more likely to engage in male typical play and less likely to engage in female typical play^41^. In addition, the rate of gender dysphoria is significantly increased when comparing natal females with congenital adrenal hyperplasia to unaffected natal females, at 3.0% and ~0.2% respectively^2,27,41^. When considering natal males, there is less available data, but there have been case reports in which natal males expressing gender dysphoria were later diagnosed with congenital hypogonadotropic hypogonadism, a condition in which levels of endogenous androgens are significantly diminished throughout development and adult life^22,43^. Given the above data, the conservation of mammalian estrogenic metabolic pathways, as well as the corollaries between sex-specific developmental and behavioral patterns, we believe that evaluation of variants of genes associated with these developmental patterns constitute one initial avenue for exploring the origins of both gender dysphoria and gender identity in general.

### The medial preoptic area

In general, ER activation during neurodevelopment results in sex specific differences in brain volume or dendritic spine density, but notably, both the intermediary pathways occurring after ER activation *and* the final effects on brain volume and density are specific to each region^33^. Within the mPOA, ER activation causes increased activity of cyclooxygenase-2 leading to increased levels of presynaptic prostaglandin-E2. Prostaglandin-E2 is released from the presynaptic neuron, where postsynaptic prostaglandin receptor activation, in conjunction with astrocyte signaling, leads to increased mobilization and activation of dendritic AMPA-type glutamate receptors, resulting in increased dendritic spine formation^28^. In rodents, increased number and density of dendrites in the mPOA is associated with male pattern behavior at maturity (i.e. mounting and intromission)^33^. As mentioned above, when AMPA receptor agonists were given exogenously to neonatal females during the 10-day critical period after birth, this resulted in adult females that demonstrated male pattern sexual behavior. In total, nine candidate genes with confirmed variants (by Sanger sequencing) were identified that could affect this pathway^44^. One gene, *SPHK1*, has been shown to modulate cyclooxygenase-2 acetylation in neurons, another, *DNER*, has been shown to mediate neuron-glia interaction, and seven others, *CDH8, CTNNA2, DSCAML1, EGF, EFHD2, SYNPO*, and *TNN*, have been implicated in dendritic formation, arrangement, and arborization (Table 4)^32,45–52^.

### The ventromedial nucleus

In the VMN, stimulation of ER on presynaptic neurons results in presynaptic release of glutamate, which in turn stimulates postsynaptic NMDA receptors. Activation of these receptors causes an influx of calcium into the neuron and activation of MAP kinase, leading to an increased number of dendritic spines in the VMN^33^. As in the mPOA, increased number and density of dendrites is associated with male pattern behavior in animal models. Four Sanger confirmed variants were noted in genes associated with this pathway. *RIMS3* and *RIMS4* both play an important role in presynaptic vesicle fusion and neurotransmitter release. *GRIN1* is an NMDA receptor and *MAP4K3* is a MAP kinase, both of which are expressed in the brain (Table 4)^53–55^.

### The anteroventral periventricular nucleus

In the AVPV, ER activation leads to increased levels of TNF-α, which thereby decreases BCL-2 activity and increases levels of BAX/BAD. This shift leads to neuronal apoptosis and lower overall AVPV volume in males. Decrease in AVPV volume is thought to prevent adult male rodents from exhibiting the LH surge characteristic of females^33^. One confirmed candidate gene, *BOK*, has been associated with the apoptosis response to BAX/BAD (Table 4)^56^.

### The arcuate nucleus

In the arcuate nucleus, neuronal ER activation during development leads to increased levels of the activated chloride channel pNKCC1, leading to an influx of Cl^–^ into the neuron^31^. This has a polarizing effect, due to the resulting Cl^–^ concentration gradient across the neuronal cellular membrane, with the higher concentration now present within the neuron. GABAAR is a bidirectional Cl^–^ channel that is also present on the cellular membrane of developing neurons^30^. When activated by GABA, GABAAR allows Cl^−^ to flow down its concentration gradient toward equilibrium, resulting in a neuronal depolarization that in turn opens L-type voltage-gated calcium channels, allowing the influx of calcium into the neuron. Potentially via calcium activated calmodulin kinase or mitogen activated protein kinase, this influx of calcium causes intraneuronal CREB to be phosphorylated to pCREB^57^. Through a still poorly understood mechanism, increased intraneuronal pCREB leads to decreased dendritic spine number in the arcuate nucleus of the developing male rat^29,33^. One confirmed candidate gene, *KCNK3*, transcribes a transmembrane K+ channel that has been shown to be upregulated in a compensatory fashion in neurons where GABAAR activity was experimentally disrupted (Table 4)^58^.

### Variant effects in transgender males compared to transgender females

Given that gender dysphoria in male and female individuals are generally considered to be distinct phenotypes, we felt that it was important to not only analyze the combined results of all enrolled individuals but also to analyze the results when considering transgender males and transgender females separately. It has been shown that ER activation has differing and sometimes opposing developmental effects in different brain regions and in different sexes. This leaves open the possibility that genes associated with those developmental pathways could contribute to gender dysphoria in both natal sexes. Along those lines, one variant of interest is *DIAPH2*: c.G736T (p.G246X), which was heterozygous in three transgender males and hemizygous in one transgender female. *DIAPH2* is located on the X chromosome and disruption of this gene has been associated with premature ovarian failure in natal females^59^.

### Other ACMG class 3 and 4 variants

In addition to the candidate genes noted above, WES revealed multiple other unique ACMG class 3 and 4 variants that we could not link to known pathways leading to sexual dimorphism in the brain. We felt that it was important to include these variants for two reasons. First, as previously mentioned, the genetic contributors and neurodevelopmental pathways contributing to gender identity are, as of yet, poorly understood. Second, there have been many cases in which new and unexpected functions have been discovered in genes with seemingly unrelated activity described previously. Though only candidate genetic variants were confirmed by Sanger Sequencing, each of the genes listed throughout this article was suggested by WES to have a variant that was not present in either the ExAC or dbSNP databases, and was also not present in 88 in-house non-transgender controls.

### Special considerations in gender identity genetic research

Several factors add layers of complexity to the investigation of the genetic contribution to gender dysphoria and transgender identity. First, like many human traits, gender identity is unlikely to result from the variation of a single gene. Notably, even the binary categories of “transgender male” and “transgender female” are not sufficient to describe all members of the gender-expansive community, with some individuals for example self-describing as gender non-binary or agender. The broad spectrum that characterizes human gender identity suggests that, rather than being tied to variation within a single gene, an individual’s gender identity is more likely the result of a complex interplay between multiple genes as well as environmental and societal factors. The above concept, in which variation of multiple genes can additively contribute to the final formation of complex human traits, is termed the Polygenic Threshold Model, and it has been cited by prior investigators as the most likely mechanism by which gender identity develops in humans^22^. These investigators noted that this model is both “inherently destigmatizing” in its presentation of a wide phenotypic spectrum and that it contradicts the idea that a specific complement of genetic variants could be used to identify or predict an individual’s gender identity. The inherent challenge to genetic research within this framework is that, rather than approaching investigation as the search for a single so-called “transgender gene”, the investigation must rather strive to understand the complexities of gender development through the lens of genetics. This requires the acknowledgement that the genetic milieu contributing to gender identity may be completely different from one individual to the next. While, in some individuals, a single genetic variant may be sufficient to result in gender dysphoria, it does not follow that that particular variant would be necessary or sufficient to cause gender dysphoria in the population at large. It is for that reason that newer modalities of genetic research, such as genome wide association studies and whole genome/exome sequencing, are more likely to be contributive to this field in a positive way. Technologies such as these, WES in the case of our study, allow for the evaluation of patterns of genetic variation that exceed the search for individual genetic variants.

A second factor that must be considered when conducting genetic research involving the transgender community is the potential societal impact that could result from the findings. This requires not only input from experts within the field of genetics and research ethics, but importantly must also consider the attitudes, opinions, and beliefs that exist within the transgender community. Of note, one of the primary reasons that we began this study was in response to inquiries by transgender patients from our clinics. Surprisingly, upon searching for formalized studies assessing these views, we were unable to find any. For that reason we have now begun a study, which is now in the early stages of data collection, precisely to assess those values. As should be true in all fields of research, but even more crucially in this area, investigators must recognize that the reporting of their findings must be made with great care and with precise language so that the results cannot be misconstrued, exaggerated, or used to negatively impact the transgender community at large.

Complicating each of the above issues is a third challenge inherent to research involving the genetic contribution to transgender identity. In general, genetic research focuses on the etiologies of pathologic traits. Because of this, the terminology currently accepted for description of genetic variants reflects an assumption that a trait caused by a potential variant is pathologic. Of note, the World Health Organization no longer classifies transgender identity as a pathologic trait, stating that the “evidence is now clear that [gender dysphoria] is not a mental disorder^60,61^”. When investigating the etiology of a trait that we do not consider to be pathologic, such as transgender identity, extreme care must be taken in the descriptions of candidate variants and their potential relation to that phenotype. For that reason, though we do utilize the American College of Medical Genetics recommendations for the description of variants, we use this terminology with the understanding that terms such as “deleterious”, “pathogenic”, or “likely pathogenic” simply describe the predicted functional effect that a particular variant will have when compared to wild-type, rather than the phenotype associated with that change. And though a full discussion of this topic is outside the scope of this article, we feel that it is worthwhile to consider the potential benefits of adopting a modified version of the current variant classification system, that could be utilized in genetic studies evaluating transgender identity and other non-pathologic states. Similar to the current classification system, the modified system would allow investigators to convey varying degrees of likelihood that a functional genetic change would be imbued by a particular variant, but without the inherently negative connotation associated with terms such as “pathogenic”.

### Limitations of this study

A primary limitation of this study was that it included only 30 subjects, though this does constitute a larger sample size than the majority of prior studies utilizing WES to study gender dysphoria. It is for that reason that we consider the above findings to be preliminary in nature. We acknowledge that for any conclusions to be drawn regarding the extent to which a specific genetic variant contributes to gender dysphoria, segregation and *in-vitro* analysis will be essential. However, we felt that it was important to report this preliminary data to provide a new framework (i.e. consideration of variants affecting sexually dimorphic brain development) for gender identity research. We continue to enroll new patients and will continue to report significant findings as they become available.

In addition, we are unable to characterize the extent of the majority of subjects’ transition processes, as this information was not collected as part of the enrollment process. However, we did make certain that each subject met the clinical criteria for gender dysphoria before enrollment. Moving forward, we may include questions assessing the timing and extent of transition as part of the enrollment process. Finally, this study was limited in that whole exome, rather than whole genome, sequencing was utilized to identify variants. This was primarily due to cost, and may be addressed in the future as the cost of whole genome sequencing continues to fall.

In summary, our study has identified genetic variants in 19 candidate genes that may be involved in pathways of gender development in the brain. These variants, first identified through WES, were then confirmed with Sanger sequencing, and each was categorized as class 3 or 4 using the ACMG classification system. None were found in the ExAC or dbSNP databases, or in 88 non-transgender controls, suggesting that they are not common polymorphisms. The new candidate genes have each been shown to have some relation to key contributors to the currently known pathways of sex-specific brain development in rodent models. Though these neurodevelopmental pathways have not been characterized in humans to the degree that they been described in animals, we believe that genes involving these pathways constitute a reasonable avenue for investigation into the genetic contribution to gender dysphoria in humans.

## Supplementary information

Supplemental Tables & Figure

## Data Availability

All data generated or analyzed during this study are included in this published article (and its Supplementary Information File).
