# Supplementary material for: The Use of Whole Exome Sequencing in a Cohort of Transgender Individuals to Identify Rare Genetic Variants
Source: Sci Rep. 2019 Dec 27;9:20099. doi: 10.1038/s41598-019-53500-y (PMC6934803; doi:10.1038/s41598-019-53500-y)
Supplement: Supplementary file 1 — Supplemental Tables & Figure [file 41598_2019_53500_MOESM1_ESM.pdf]

Evaluating Rare Genetic Variants Identified by Whole Exome Sequencing in a Cohort of Transgender Individuals

Authors: J. Graham Theisen, Viji Sundaram, Mary S. Filchak, Lynn P. Chorich, Megan E. Sullivan, James Knight, Hyung-Goo Kim, Lawrence C. Layman

A marked incongruence between one's experienced/expressed gender and natal gender of at least 6 months in duration, as manifested by at least two of the following:
A marked incongruence between one's experienced/expressed gender and primary and/or secondary sexual characteristics
A strong desire to be rid of one's primary and/or secondary sexual characteristics because of a marked incongruence with one's experienced/expressed gender
A strong desire for the primary and/or secondary sexual characteristics of the other gender
A strong desire to be of the other gender
A strong desire to be treated as the other gender
A strong conviction that one has the typical feelings and reactions of the other gender

Supplemental Table 1: DSM-V Diagnostic Criteria for Gender Dysphoria

Evaluating Rare Genetic Variants Identified by Whole Exome Sequencing in a Cohort of Transgender Individuals

Authors: J. Graham Theisen, Viji Sundaram, Mary S. Filchak, Lynn P. Chorich, Megan E. Sullivan, James Knight, Hyung-Goo Kim, Lawrence C. Layman

Gene	Genomic location	Position^	Wild-Type	Variant	Het◊ (n)	Hom□ (n)
ABCA12	2q35	215866455	A	G	1	0
ACAP1	17p13.1	7252464	C	T	1	0
ACVR1B	12q13.13	52370254	C	T	1	0
ADAMTS13	9q34.2	136291419	A	G	1	0
ADGRV1	5q14.3	89948215	T	C	1	0
AHCTF1	1q44	247030568	T	C	1	0
AHSA2	2p15	61412695	T	G	1	0
AKR1C3	10p15.1	5144369	A	G	1	0
AKT1S1	19q13.33	50376378	G	A	1	0
AP1B1	22q12.2	29747207	C	T	1	0
APBB1IP	10p12.1	26825142	C	A	3	0
ARHGAP40	20q11.23	37272368	A	G	1	0
ARHGEF17	11q13.4	73020830	A	T	1	0
ARMC12	6p21.31	35716397	T	A	1	0
ASMT	Xp22.33	1734120	C	T	0	1*
ATP5J2-PTCD1	7q22.1	99022588	A	G	1	0
ATRN	20p13	3584887	T	C	1	0
ATRX	Xq21.1	76952091	T	A	1	0
BAMBI	10p12.1	28971203	T	A	1	0
BAZ2B	2q24.2	160295537	G	A	1	0
BOK	2q37.3	242501871	C	T	1	0
BRD4	19p13.12	15366150	G	A	1	0
BTBD17	17q25.1	72353787	G	A	1	0
C19orf67	19p13.12	14194360	C	T	1	0
C2CD3	11q13.4	73849935	T	A	1	0
C2CD4D	1q21.3	151811152	G	A	1	0
C7	5p13.1	40947795	G	C	1	0
CAPN2	1q41	223931837	A	G	1	0
CCDC14	3q21.1	123680116	C	T	1	0
CCDC141	2q31.2	179912087	A	G	1	0
CCDC168	13q33.1	103381950	G	A	1	0
CCL18	17q12	34397891	G	T	1	0
CCSER1	4q22.1	91549362	G	C	1	0
CDC42BPG	11q13.1	64595038	C	T	1	0
CDH8	16q21	61858936	T	C	1	0
CDK12	17q12	37618361	G	C	1	0
CHD3	17p13.1	7797514	G	T	1	0
CILP	15q22.31	65489439	G	A	1	0
CKAP2	13q14.3	53049199	G	A	1	0
COG5	7q22.3	106938734	T	C	1	0

COL4A4	2q36.3	227953499	G	A	1	0
COL4A4	2q36.3	227872163	A	G	1	0
COL5A1	9q34.3	137534115	C	A	1	0
CPQ	8q22.1	97892144	A	T	1	0
CSDC2	22q13.2	41968123	A	G	1	0
CSRNP1	3p22.2	39185154	C	T	1	0
CTNNBL1	20q11.23	36405783	A	G	1	0
CXorf57	Xq22.3	105876423	C	T	0	1
DDX17	22q13.1	38897221	G	A	1	0
DEFB129	20p13	209997	A	G	1	0
DGCR6L	22q11.21	20366867	G	T	1	0
DNAH14	1q42.12	225586760	C	T	1	0
DNAH3	16p12.3	21042566	T	C	1	0
DNAH3	16p12.3	21042508	C	G	1	0
DNAH6	2p11.2	85008699	G	T	1	0
DNER	2q36.3	230578982	C	A	1	0
DOT1L	19p13.3	2223379	C	T	1	0
DSCAM	21q22.2	41414573	G	T	3	0
DSCAM	21q22.2	41414571	C	T	3	0
DSCAM	21q22.2	41710222	T	G	1	0
DSCAML1	11q23.3	117340673	C	T	1	0
EFHD2	1p36.21	15753774	G	T	1	0
EGF	4q25	110890223	G	C	1	0
EHD4	15q15.1	42235310	C	T	1	0
EYS	6q12	66204756	C	A	1	0
F13B	1q31.3	197026454	C	T	1	0
FAM184B	4p15.32-p15.31	17711264	T	A	1	0
FAM19A4	3p14.1	68802062	G	C	1	0
FAM21C	10q11.22	46252504	G	C	1	0
FBLN2	3p25.1	13649667	T	A	1	0
FCGBP	19q13.2	40383820	C	T	2	0
FCGBP	19q13.2	40383904	C	T	1	0
FCGBP	19q13.2	40366204	G	A	1	0
FLNC	7q32.1	128489556	C	T	1	0
FLRT2	14q31.3	86089168	T	A	1	0
FSTL5	4q32.2	162459410	T	G	1	0
FYCO1	3p21.31	46008362	C	G	1	0
GAL3ST3	11q13.1	65810801	A	G	1	0
GFI1B	9q34.13	135865217	G	A	1	0
GLDC	9p24.1	6587253	G	C	1	0
GOLGA8J	15q13.2	30381959	C	G	2	0

GOLGA8K	15q13.3	32685713	T	C	1	0
GOLGA8M	15q13.1	28947450	T	C	1	0
GPR63	6q16.1	97247010	C	T	1	0
GPR85	7q31.1	112724668	C	G	1	0
GRIN1	9q34.3	140036549	C	G	1	0
GSC	14q32.13	95236291	G	C	1	0
GTPBP6	Xp22.33	224532	C	A	0	1*
GTPBP6	Xp22.33	228205	C	T	1	0
H2AFY2	10q22.1	71835533	G	A	1	0
HCN3	1q22	155252457	C	G	1	0
HCN4	15q24.1	73622060	C	T	1	0
HHIPL1	14q32.2	100125875	C	G	1	0
HMCES	3q21.3	129023538	C	T	1	0
IGLL1	22q11.23	23922357	C	G	1	0
IQSEC3	12p13.33	271157	A	C	1	0
IRS2	13q34	110435525	C	T	1	0
KCNG2	18q23	77659592	G	A	1	0
KCNK3	2p23.3	26950997	T	C	1	0
KDM4E	11q21	94758794	T	C	1	0
KHNYN	14q12	24900976	C	T	1	0
KIAA0586	14q23.1	58927786	G	A	1	0
KIAA1107	1p22.1	92647175	T	C	1	0
KIAA1109	4q27	123192588	A	C	1	0
KIAA1549L	11p13	33667512	G	A	1	0
KIF19	17q25.1	72348048	C	G	1	0
KMT2D	12q13.12	49427026	T	A	1	0
KRTAP1-5	17q21.2	39182996	A	G	1	0
LAMA2	6q22.33	129591785	C	T	1	0
LAMB1	7q31.1	107615774	T	C	1	0
LARP1	5q33.2	154181721	C	T	1	0
LETM1	4p16.3	1816276	C	A	1	0
LGI3	8p21.3	22005920	G	A	1	0
LGR6	1q32.1	202266673	G	A	1	0
LRBA	4q31.3	151356772	C	T	1	0
MAGI3	1p13.2	113933885	C	G	1	0
MAP4K3	2p22.1	39499479	G	C	1	0
MDM1	12q15	68707490	T	C	1	0
METTL25	12q21.31	82780626	T	G	1	0
MFAP4	17p11.2	19289660	A	G	1	0
MFSD6L	17p13.1	8700983	G	A	1	0
MTFR1	8q13.1	66620183	C	G	1	0

NAT1	8p22	18080344	T	C	1	0
NCKAP1	2q32.1	183817934	T	A	1	0
NCKAP5L	12q13.12	50186581	C	T	1	0
NDST3	4q26	119064715	T	G	1	0
NFAM1	22q13.2	42783044	T	C	1	0
NFKBIZ	3q12.3	101571995	A	C	1	0
NGDN	14q11.2	23946539	A	G	1	0
NLRP3	1q44	247587601	C	A	1	0
NOL12	22q13.1	38083977	G	C	1	0
NPIPB3	16p12.2	21416089	C	T	1	0
NR1D2	3p24.2	24001229	C	A	1	0
NSUN5P2	7q11.23	72436394	A	G	1	0
NUDT19	19q13.11	33183507	C	T	1	0
OGFR	20q13.33	61443743	T	C	1	0
OR2T12	1q44	248458721	G	A	1	0
OR5A1	11q12.1	59211124	C	G	1	0
OTOG	11p15.1	17667311	T	G	1	0
P2RY8	Xp22.33	1585099	C	G	0	1*
PATL1	11q12.1	59423514	G	A	2	0
PDE3A	12p12.2	20803464	A	G	1	0
PDE8B	5q13.3	76721613	G	A	1	0
PFN3	5q35.3	176827225	C	G	1	0
PGC	6p21.1	41704612	A	G	1	0
PGM2L1	11q13.4	74049623	G	T	1	0
PLBD2	12q24.13	113812705	T	C	1	0
PLCXD1	Xp22.33	208284	T	C	0	1*
PLCXD1	Xp22.33	207345	G	A	0	1*
PLEC	8q24.3	145001865	G	C	1	0
PLEKHG2	19q13.2	39908575	G	A	1	0
POLQ	3q13.33	121209209	G	A	1	0
PPARGC1B	5q32	149213068	C	T	1	0
PPP2R3B	Xp22.33	299424	C	T	1	0
PRAMEF7	1p36.21	12980073	G	T	1	0
PRRC2C	1q24.3	171509504	G	A	1	0
PTPRR	12q15	71286493	T	C	1	0
RASA4B	7q22.1	102125585	C	T	3	0
RGAG1	Xq23	109696838	C	G	0	1*
RIMS3	1p34.2	41107405	T	C	1	0
RIMS4	20q13.12	43384843	G	A	1	0
RLF	1p34.2	40697193	C	G	1	0
ROCK2	2p25.1	11341207	C	T	1	0

RTEL1	20q13.33	62321182	G	A	1	0
RYR3	15q13.3-q14	33825537	G	A	1	0
SDK2	17q25.1	71443796	C	T	1	0
SDK2	17q25.1	71357877	T	C	1	0
SETBP1	18q12.3	42532819	G	C	1	0
SGPP2	2q36.1	223289368	G	T	1	0
SHPRH	6q24.3	146273568	A	G	1	0
SIN3B	19p13.11	16976363	G	A	1	0
SLC18A1	8p21.3	20038420	G	T	1	0
SLFN14	17q12	33875323	G	A	1	0
SPAG9	17q21.33	49074033	G	A	1	0
SPHK1	17q25.1	74383224	C	T	1	0
SPRN	10q26.3	135237026	C	G	1	0
SRRM3	7q11.23	75864530	G	A	1	0
SSC5D	19q13.42	56001745	C	A	1	0
STARD9	15q15.2	42983286	T	G	1	0
STRA6	15q24.1	74494529	T	G	1	0
SYNPO	5q33.1	150028774	G	A	1	0
SYNPO2L	10q22.2	75408070	G	A	1	0
TBC1D3F	17q12	34581905	A	G	1	0
TBC1D5	3p24.3	17349600	C	T	1	0
TCFL5	20q13.33	61492458	C	T	1	0
TDRD12	19q13.11	33301036	T	C	1	0
TDRD6	6p12.3	46656668	A	G	1	0
THEG	19p13.3	374349	C	A	1	0
TMPRSS6	22q12.3	37462272	A	G	1	0
TMPRSS9	19p13.3	2425456	G	A	1	0
TNN	1q25.1	175046693	A	G	1	0
TNRC18	7p22.1	5427886	C	T	1	0
TOPBP1	3q22.1	133336050	G	C	1	0
TRAPPC9	8q24.3	141321451	G	C	1	0
TTF1	9q34.13	135261962	C	T	1	0
UBR4	1p36.13	19415318	G	A	1	0
UNC13C	15q21.3	54630632	C	T	1	0
USH2A	1q41	215814050	C	T	1	0
VAV2	9q34.2	136649442	A	C	1	0
VPS11	11q23.3	118949558	T	G	1	0
WNT11	11q13.5	75898144	A	G	1	0
ZBTB7C	18q21.1	45555662	T	C	1	0
ZC3H7B	22q13.2	41742112	G	A	1	0
ZC3HAV1	7q34	138793885	C	T	1	0

ZFHX4	8q21.13	77765857	A	C	1	0
ZHX2	8q24.13	123964676	G	T	1	0
ZNF132	19q13.43	58945166	C	T	1	0
ZNF276	16q24.3	89789805	T	G	1	0
ZNF311	6p22.1	28963376	T	C	1	0
ZNF492	19p12	22847609	C	T	1	0
ZNF512	2p23.3	27826034	A	T	1	0
ZNF578	19q13.41	53014748	A	G	1	0
ZNF652	17q21.33	47390063	C	T	1	0
ZNF699	19p13.2	9407313	C	T	1	0
ZNF785	16p11.2	30594038	G	A	1	0
ZNF800	7q31.33	127013411	G	C	1	0
ZSCAN10	16p13.3	3139155	G	T	1	0
ZSCAN5A	19q13.43	56733120	G	T	1	0
ZXDA	Xp11.21	57936299	G	A	1	0

Supplemental Table 2: Missense Variants Called by Whole Exome Sequencing

^Position based on GRCh37.p13 Primary Assembly

◊Heterozygous

□Homozygous

*Reported as hemizygous in a transgender female subject

The Use of Whole Exome Sequencing in a Cohort of Transgender Individuals to Identify Rare Genetic Variants

J. Graham Theisen, Viji Sundaram, Mary S. Filchak, Lynn P. Chorich, Megan E. Sullivan, James Knight, Hyung-Goo Kim, Lawrence C. Layman

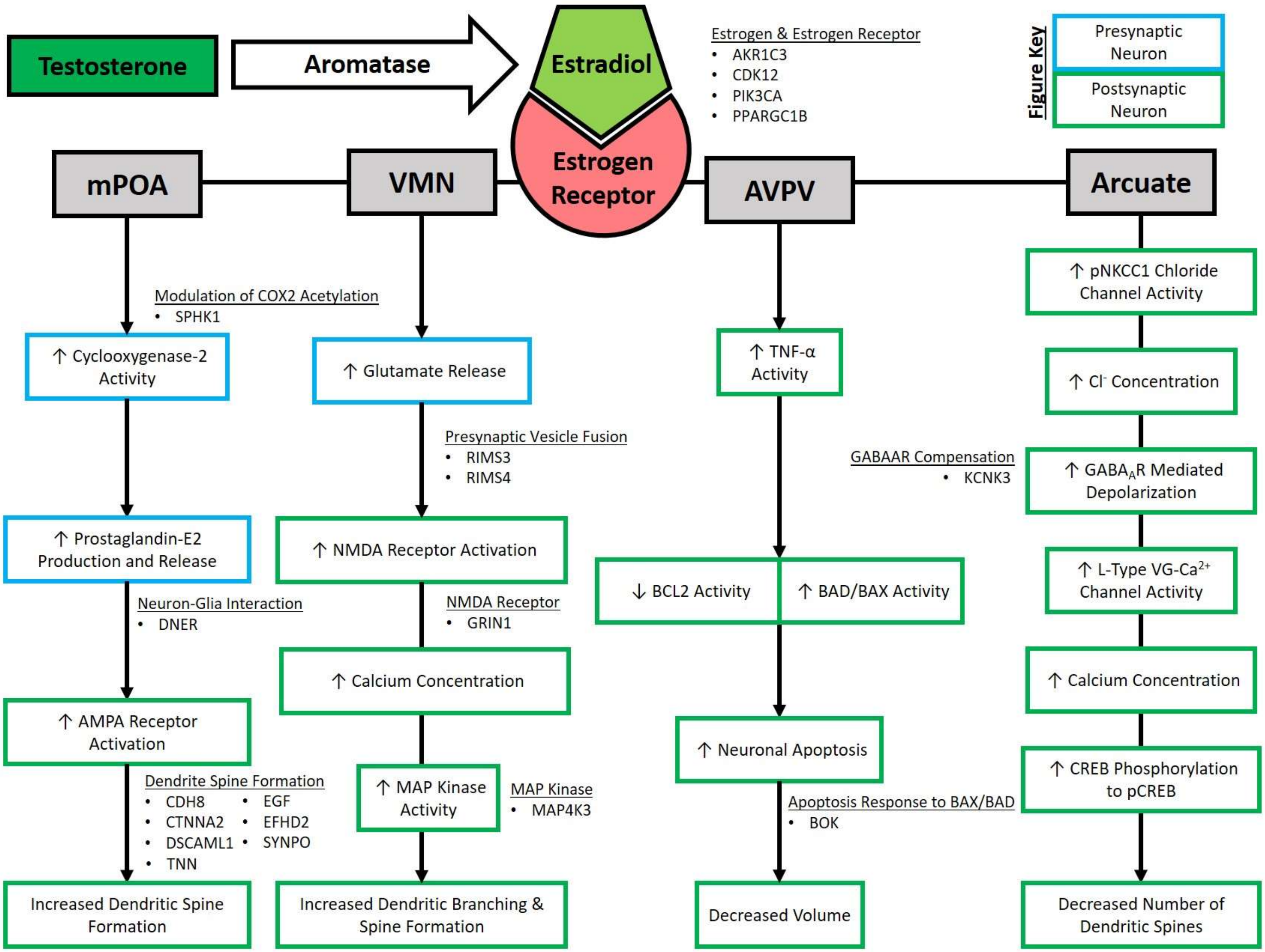

Supplemental Figure 1: Diagram of estradiol receptor activated neurodevelopmental pathways in sexually dimorphic brain regions, including the ventromedial nucleus (VMN), the medial preoptic area (mPOA), the anteroventral periventricular nucleus (AVPV), and the arcuate nucleus. Candidate genes are listed where their descriptions, functions, or ontologies suggested a potential association with these pathways.
